# Impact of time to full enteral feeding on long-term neurodevelopment without mediating by postnatal growth failure in very-low-birth-weight-infants

**DOI:** 10.1038/s41598-023-29646-1

**Published:** 2023-02-20

**Authors:** Shin Ae Yoon, Myung Hee Lee, Yun Sil Chang

**Affiliations:** 1 Department of Pediatrics, Chungbuk National University Hospital, Chungbuk National University School of Medicine, 1 Sunhwan ro 776, Seowon-gu, Cheongju, 28644 Republic of Korea; 2 Research and Statistical Center, Social Information Research Institute, Seoul, Republic of Korea; 3 Department of Pediatrics, Samsung Medical Center, Sungkyunkwan University School of Medicine, 81 Irwon-Ro, Gangnam-Gu, Seoul, 06351 Republic of Korea; 4 Department of Health Sciences and Technology, SAIHST, Sungkyunkwan University, 81 Irwon-Ro, Gangnam-Gu, Seoul, 06351 Republic of Korea; 5 Samsung Medical Center, Cell and Gene Therapy Institute, Seoul, Republic of Korea; 6 MEDITOS, Institute of Biomedical and Clinical Research, Seoul, Republic of Korea

**Keywords:** Health care, Medical research, Risk factors

## Abstract

This study aimed to determine if time to achieve full enteral feeding (TFF) directly impacted long-term neurodevelopmental delay (NDD) and whether long-term postnatal growth failure (PGF) was a mediator of this association in very-low-birth-weight (VLBW) infants. Using prospectively collected cohort data from the Korean Neonatal Network, we included eligible VLBW infants who achieved TFF at least once and classified enrolled infants into four groups using exposure severity (P1 to P4 as TFF < 16, 16–30, 31–45, and > 45 postnatal days, respectively). After adjusting for confounding variables, survival without NDD was significantly decreased in P4 infants compared with that in P2 infants. P1 infants had a lower risk of weight and height PGF than P2 infants; however, P4 infants had higher risks of height and head circumference PGF than P2 infants. Weight and height PGF were significantly associated with an increased risk of NDD. In mediation analysis, early and delayed TFF revealed direct positive and negative impacts, respectively, on the risk of NDD without mediation by PGF. TFF impacted survival without NDD, and PGF did not mediate this association in VLBW infants. Additionally, these results can be translated into evidence-based quality improvement practice.

## Introduction

Achieving full enteral feeding in very-low-birth-weight (VLBW) infants implies a successful transition from parenteral to enteral feeding and the establishment of optimal postnatal nutrition during neonatal intensive care. Therefore, time to achieve full enteral feeding (TFF) in VLBW infants is an important clinical course index targeted for quality care^1,2^. Delayed TFF is related to postnatal growth failure (PGF)^3–5^, prolonged hospital stay^3^, and poor brain growth^6^. Therefore, various attempts have been made to achieve early TFF without increasing the risk of necrotizing enterocolitis (NEC), such as determining optimal timing to initiate postnatal enteral feeding or safely increase enteral feeding volume in VLBW infants^7–10^. Nevertheless, many neonatologists have doubts regarding the direct impact of TFF on long-term growth and neurodevelopment of VLBW infants beyond in-hospital mortality and morbidities^3,5,6^. In addition, there is doubt concerning mediation of the relationship between delayed TFF and poor neurodevelopment by long-term growth. Furthermore, there are conflicting data on the relationship between TFF and long-term growth^4,5,11^, TFF and neurodevelopment^12,13^, and long-term growth and neurodevelopment^3,14–16^ in preterm infants.

In this study, we hypothesized that TFF would be associated with long-term survival without neurodevelopmental delay (NDD) among VLBW infants as the primary outcome as well as decreased risk of long-term PGF as the secondary outcome. In addition, we hypothesized that decreased long-term PGF mediates the relationship between the early achievement of full enteral feeding and improved survival without NDD.

To test this hypothesis, we categorized VLBW infants who achieved full enteral feeding at least once according to TFF using the large national cohort of the Korean Neonatal Network (KNN)^17^.We evaluated and compared long-term neurodevelopmental and growth outcomes at 18–24 months’ corrected age (CA) according to TFF adjusted with significant confounders derived from their basal characteristics and short-term morbidities. Then, we finally conducted mediation analysis of the effect of TFF on NDD when mediated by PGF at CA 18–24 months.

## Materials and methods

### Study design and data source

We performed this cohort study using a deidentified data set of KNN, approved by the Committee of Ethics and Publication of the KNN, extracted from the internet-based clinical trial management system (i-CREAT) of the Korea National Institute of Health. The KNN registry comprised VLBW infants (birth weight < 1500 g) admitted to the corresponding neonatal intensive care unit (NICU) at birth or transferred from other hospitals within 28 days after birth. Clinicians in over 60 KNN-participating NICUs prospectively collected data regarding perinatal and neonatal information (growth and neurodevelopment) at discharge, during NICU hospitalization, and at 18–24 months’ CA via pre-set KNN manual of operation (MOP) using electronic case report forms based on the i-CREAT system. These KNN registry data are annually stored in the Korea National Institute of Health server after completing data quality management using queries and site-visit monitoring^17^. The KNN registry was approved by the Institutional Review Board at each participating hospital, and informed consent was obtained from parents at enrollment in NICUs participating in the KNN. We confirm that all methods were performed in accordance with the relevant guidelines and regulations.

### Study population

We identified all VLBW infants registered in the KNN born between January 1, 2013 and December 31, 2015. We excluded infants born at < 23 or > 32 weeks of gestation or infants having major congenital anomalies, including lethal or life-threatening birth defects requiring repair surgery or immediate intervention. According to the KNN MOP, TFF was defined as postnatal days (PNDs) when the enteral feeding volume initially reached 100 mL/kg/day of milk regardless of milk type, usually when the intravenous nutrition stopped in most centers. Therefore, we excluded infants who died without achieving full enteral feeding, those without TFF during their NICU stay, or infants with incomplete TFF data.

Due to the skewed TFF distribution of the enrolled cohort, when determining the exposure severity by dividing TFF interval, we excluded data points that were more than 1.5 times the interquartile range (IQR) above the third quartile according to the 1.5 IQR rule^18^. Based on the preliminary analysis, we categorized eligible VLBW infants into four groups based on TFF using 15-day increments: P1, < 16 PND; P2, 16–30 PND; P3, 31–45 PND; and P4, > 45 PND.

### Data collection

Perinatal, neonatal, and long-term follow-up data for each infant at 18–24 months’ CA were obtained. Perinatal data included information on gestational age (GA), birth weight, Apgar scores at 1 and 5 min, sex, delivery mode, small-or-GA (birth weight below the 10th percentile based on the Fenton’s growth chart), ^19^ antenatal steroid use, premature membrane rupture, histological chorioamnionitis, pregnancy-induced hypertension (PIH), maternal diabetes mellitus, and social determinants, such as race and parents’ education level. In addition, neonatal data included information on intraventricular hemorrhage (IVH) (≥ grade 3)^20^, culture-proven sepsis, NEC (≥ Bell’s stage 2)^21^, periventricular leukomalacia (PVL)^22^, bronchopulmonary dysplasia (BPD) (≥ moderate)^23^, retinopathy of prematurity requiring laser treatment^24^, duration of parenteral nutrition and hospital stay, cause of death, and sex- and age-specific Z-scores for height, weight, and head circumference measured at birth and discharge from NICU according to the Fenton’s growth chart^19^. Furthermore, long-term data at 18–24 months’ CA included information on weight, height, and head circumference Z-scores by the World Health Organization (WHO) Child Growth Standards^25,26^, cerebral palsy status^27^, visual-sensory impairments defined as blindness or wearing eyeglasses and hearing impairments defined as bilateral impairment requiring hearing aids, and any results of tests received including implementing Bayley Scales of Infant and Toddler Development (BSID II, III), Korean version of the Ages and Stages Questionnaire (K-ASQ), and Korean-Developmental Screening Test for infants and children (K-DST). K-ASQ and K-DST are neurodevelopmental screening tools using parental questionnaires approved by the Korean Society of Pediatrics. K-DST has been nationally conducted since the late 2014 in Korea.

### Outcomes

The primary outcome was survival without NDD at 18–24 months’ CA. NDD was defined as scores < 70 according to BSID-II or III^28,29^, or when critical cut-off scores for each domain were less than 2 standard deviation (SD) under the mean value, implying referral for further assessment using the K-ASQ or K-DST^30–32^. The secondary outcomes were cerebral palsy, sensory impairments, and each domain of NDD and PGF at 18–24 months’ CA. We defined NDD by categorizing it into separate domains using related results from all available tests, namely motor, mental, and social domains (Supplementary Fig. S1 online). K-ASQ or K-DST is highly correlated with BSID-II or BSID-III, ^33,34^ however, we considered BSID-II or III as a confirmatory test and K-ASQ or K-DST as a screening test for NDD (Supplementary Table S1). When any of the three domains was abnormal, it was defined as NDD for the total domain.

Regarding growth outcomes, we compared the difference in Z-score between birth and discharge and between discharge and a CA of 18–24 months. We assessed PGF at discharge and at 18–24 months’ CA. Reflecting that several healthy preterm infants are placed in PGF, conventionally below the 10th percentile on growth charts, PGF was defined as weight, height, or head circumference at each point that did not exceed a Z-score of –2 (2.3rd percentile) according to the WHO Child Growth Standards^25,35–37^, not using the changes in Z-score between two times. This was because when we examined the data distribution of the Z-score difference between 18 and 24 months’ CA and discharge, infants having a Z-score < –2 at 18–24 months’ CA were largely excluded from the condition where the Z-score difference was < –2.

### Statistical analysis

Continuous variables were presented as means ± SD and compared using ANOVA with post hoc test (Scheffe) or Kruskal–Wallis test with post hoc test (Dunn) designed to control the family wise error rate according to Bartlett’s test of sphericity results^38^. Categorical variables were presented as percentages and frequencies and compared using the chi-squared or Fisher’s exact test. A modified Poisson regression with a robust error variance and a log link treating violations of Poisson distribution assumption for common binary outcomes were performed using a generalized linear model to estimate the relative risk (RR)^39^. It estimated the adjusted RR for potential confounders with 95% confidence intervals (CIs). Average TFF in this cohort was 24 PNDs, falling into P2 group. Therefore, RRs for primary and secondary outcomes of each group at 18–24 months’ CA were compared with reference to P2 group. Furthermore, impact of PGF on NDD at 18–24 months’ CA was evaluated using univariate analysis, multiple logistic regression analysis, and a forest plot. Mediation analysis was performed using the R package, medflex for estimating direct natural effects and natural indirect effects through the natural effect model proposed by Lange et al.^40^ and Vansteelandt et al.^41^ All statistical analyses were carried out using Stata SE version 14.2 (Stata Corp.) and R version 4.1.2. All statistical tests were two-tailed, and *P-*values < 0.05 were considered statistically significant.

## Results

### Demographics and short-term outcomes

**Figure 1:**
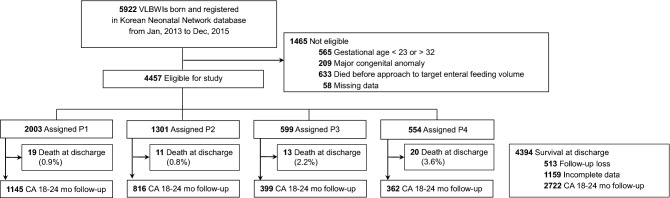
Flowchart identifying the study population. We categorized these eligible infants into four groups based on the time to reach full enteral feeding by 15 days increment: P1, < 16 postnatal days (PND); P2, 16–30 PNDs; P3, 31–45 PNDs; and P4, > 45 PNDs. VLBWI, Very-low-birth-weight infants; CA, corrected age.

Initially, 5922 VLBW infants were identified, of which 1465 infants were excluded using the exclusion criteria. Of the remaining infants, 63 died before NICU discharge, 513 were lost to follow-up, and 1159 had incomplete data at 18–24 months’ CA. Subsequently, the 4457 infants included were classified based on TFF into four groups using the short-term data. Among them, 2722 infants (61.9%) had long-term data at 18–24 months’ CA (Fig. 1).

Demographic characteristics and short-term outcomes before NICU discharge are described in Supplementary Table S2. Mean GA and birth weights were 28^5/7^ ± 2.2 weeks and 1093.1 ± 261.4 g, respectively. Mean TFF was 24 ± 20 days. In addition, mean GA, birth weight, and Apgar scores significantly decreased in infants from P1 to P4. The rate of antenatal steroid use and histological chorioamnionitis increased and PIH incidence in infants decreased from P1 to P4. The incidence of morbidities, such as high-grade IVH, sepsis, NEC (≥ 2), PVL, BPD (moderate-to-severe), and retinopathy of prematurity requiring laser therapy, increased significantly in infants from P1 to P4. Mortality increased in infants from P2 to P4. In addition, there were no differences regarding causes of death among groups (*P* = 0.946); however, PND at death increased significantly from P1 to P4 (*P* < 0.001).

### Primary and secondary outcomes at 18–24 months’ corrected age

Most parameters representing NDD from BSID-II, BSID-III, K-DST, and K-ASQ tests increased significantly in infants from P1 to P4. In addition, the incidence of cerebral palsy and sensory impairments significantly increased in infants from P1 to P4 (Supplementary Table S3).

We included all significant variables, which are well-known significant factors affecting long-term outcomes^42^, in the model to derive adjusted RRs for primary and secondary outcomes of P1, P3, and P4 infants than those of P2 infants.

Among all infants, survival without NDD significantly decreased in P4 infants (RR 0.88; 95% CI 0.81–0.95) than in P2 infants. P1 infants had significantly lower NDD risks in the total and motor domains than P2 infants (*P* = 0.030 and *P* = 0.004, respectively). In addition, P4 infants had significantly higher NDD risks than P2 infants in motor, mental, and social domains (*P* = 0.004, *P* < 0.001, and *P* = 0.019, respectively). Furthermore, P1 infants had a lower PGF risk of weight and height than P2 infants (*P* = 0.023 and *P* = 0.007, respectively). However, P4 infants had a higher PGF risk of height and head circumference than P2 infants (*P* = 0.031 and *P* < 0.001, respectively) (Table 1).

**Table 1:** Adjusted relative risk for neurodevelopmental and growth outcomes at 18–24 months’ corrected age.

Variables	P1: ≤ 15 PNDs (n = 1145) Adjusted RR (95% CI)^a^	P2: 16–30 PNDs (n = 816)	P3: 31–45 PNDs (n = 399) Adjusted RR (95% CI)^a^	P4: ≤ 46 PNDs (n = 362) Adjusted RR (95% CI)^a^
Primary outcome				
Survival	0.99 (0.99, 1.00)	1 (Reference)	1.00 (0.99, 1.00)	0.99 (0.99, 1.00)
Survival without NDD	1.03 (0.99, 1.07)	1	0.95 (0.89, 1.01)	0.88 (0.81, 0.95)
Secondary outcome				
Cerebral palsy	0.70 (0.47**–**1.03)	1	1.04 (0.73**–**1.51)	0.91 (0.59**–**1.40)
Visual impairment	0.95 (0.74**–**1.23)	1	1.04 (0.81**–**1.33)	1.08 (0.84**–**1.39)
Deafness	0.78 (0.38**–**1.58)	1	1.41 (0.66**–**3.02)	0.84 (0.37**–**1.91)
Neurodevelopmental delay in				
Total domain	0.77 (0.59**–**0.97)		1.29 (1.03**–**1.61)	1.40 (1.12**–**1.76)
Motor domain	0.62 (0.45**–**0.86)	1	1.51 (1.16**–**1.98)	1.67 (1.26**–**2.21)
Mental domain	0.84 (0.62**–**1.15)	1	1.30 (0.97**–**1.74)	1.36 (1.02**–**1.81)
Social domain	0.74 (0.39**–**1.40)	1	2.05 (1.21**–**3.48)	1.88 (1.11**–**3.18)
Postnatal growth failure (Z-score < − 2.0)				
Weight	0.69 (0.50**–**0.95)	1	1.11 (0.80**–**1.53)	1.13 (0.81, 1.59)
Height	0.63 (0.45**–**0.88)	1	0.94 (0.65**–**1.34)	1.43 (1.03**–**1.99)
Head circumference	1.07 (0.79**–**1.45)	1	1.49 (1.10**–**2.02)	1.75 (1.28**–**2.39)

Abbreviations: PND, postnatal day; RR, relative risk; CI, confidence interval; NDD, neurodevelopmental delay.

^a^Adjusted for gestational age, Apgar score at five min, small for gestational age, antenatal steroid use, and presence of pregnancy-induced hypertension, intraventricular hemorrhage (≥ grade 3), total sepsis, periventricular leukomalacia, bronchopulmonary dysplasia (≥ moderate), necrotizing enterocolitis (≥ 2b), and retinopathy of prematurity requiring laser therapy.

### Postnatal growth and relation between postnatal growth failure and neurodevelopmental delay at 18–24 months’ corrected age

Z-scores at discharge and at 18–24 months’ CA decreased significantly in infants from P1 to P4. Z-score differences between birth and discharge significantly decreased, whereas they significantly increased between discharge and at 18–24 months’ CA in infants from P1 to P4. The PGF rate at discharge and at 18–24 months’ CA significantly increased in infants from P1 to P4 (Supplementary Table S4).

**Figure 2:**
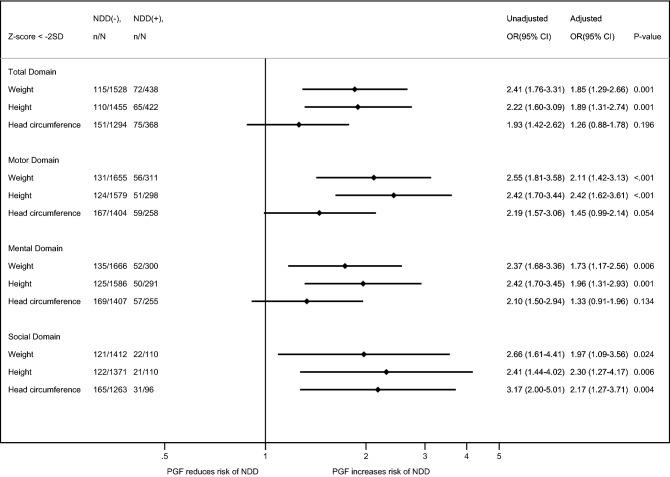
Relationship between postnatal growth failure and neurodevelopmental delay at 18–24 months’ corrected age. Abbreviations: NDD, neurodevelopmental delay; OR, odd ratio; CI, confidence intervals; PGF, postnatal growth failure (Z-score < − 2).

Additionally, infants with NDD had a significantly higher risk of height and weight PGF at 18–24 months’ CA than those without NDD in all domains, including total, motor, mental, and social domains (Fig. 2).

### Mediation between time to full enteral feeding and neurodevelopmental delay by postnatal growth failure

A mediation analysis, based on the assumption that there was no unmeasured confounding factor, was performed to investigate the association between TFF and NDD that PGF did not mediate. A Poisson regression model with a log link function was used to elucidate the presence of significant differences between the pure direct and indirect effect relative risks between the three exposure levels and P2 infants. In the analysis of pure direct effects, P1 infants had significantly decreased NDD risks compared to P2 infants, except for PGF in weight. In contrast, P3 and P4 infants had significantly increased NDD risks compared to P2 infants for PGF in all growth parameters. In the analysis of pure indirect effects, there was no significant effect of TFF on the NDD that works through PGF in each growth parameter (Table 2). Therefore, mediation analysis demonstrated the impact that TFF had on NDD, which was not mediated by each growth parameter.

**Table 2:** Mediation analysis between time to full enteral feeding and neurodevelopmental delay by postnatal growth failure.

Mediator variables	P1: ≤ 15 PNDs				P3: 31–45 PNDs					P4: ≥ 46 PNDs		
	Relative risk^a^	95% CI^b^		*P* value^c^	Relative risk^a^	95% CI^b^		*P* value^c^	Relative risk^a^	95% CI^b^		*P* value^c^
		Lower	Upper			Lower	Upper			Lower	Upper	
PGF in weight (n = 1932)												
Pure direct effect	0.786	0.608	1.012	.064	1.304	1.038	1.631	.021	1.401	1.119	1.769	.004
Total direct effect	0.779	0.607	0.996	.047	1.302	1.039	1.629	.021	1.388	1.110	1.749	.005
Pure indirect effect	0.988	0.959	1.017	.434	0.996	0.971	1.021	.740	1.008	0.972	1.046	.69
Total indirect effect	0.979	0.923	1.039	.484	0.995	0.958	1.035	.793	0.998	0.974	1.024	.90
Total effect	0.769	0.601	0.982	.036	1.297	1.032	1.626	.024	1.399	1.120	1.763	.004
PGF in height (n = 1843)												
Pure direct effect	0.741	0.571	0.954	.022	1.332	1.058	1.667	.013	1.334	1.041	1.710	.02
Total direct effect	0.768	0.592	0.988	.043	1.321	1.049	1.661	.018	1.305	1.019	1.671	.04
Pure indirect effect	0.982	0.950	1.016	.291	0.986	0.953	1.022	.427	1.033	0.982	1.088	.21
Total indirect effect	1.018	0.978	1.060	.375	0.978	0.928	1.037	.424	1.010	0.977	1.045	.55
Total effect	0.754	0.584	0.967	.028	1.302	1.036	1.640	.025	1.348	1.056	1.722	.02
PGF in head circumference (n = 1631)												
Pure direct effect	0.734	0.557	0.946	.022	1.328	1.022	1.714	.032	1.528	1.175	1.986	.002
Total direct effect	0.739	0.561	0.952	.024	1.369	1.061	1.757	.015	1.477	1.133	1.923	.004
Pure indirect effect	1.002	0.986	1.018	.853	1.009	0.958	1.064	.743	1.013	0.939	1.096	.74
Total indirect effect	1.008	0.986	1.031	.473	1.040	0.989	1.097	.133	0.979	0.930	1.035	.45
Total effect	0.740	0.562	0.953	.025	1.381	1.074	1.770	.011	1.497	1.157	1.941	.002
All PGF (n = 1935)												
Pure direct effect	0.752	0.584	0.962	.025	1.265	1.008	1.587	.043	1.417	1.125	1.792	.003
Total direct effect	0.768	0.595	0.981	.038	1.277	1.023	1.598	.031	1.355	1.078	1.711	.01
Pure indirect effect	0.990	0.961	1.021	.516	1.011	0.977	1.047	.521	1.037	0.981	1.095	.20
Total indirect effect	1.011	0.972	1.050	.590	1.022	0.976	1.070	.365	0.992	0.953	1.032	.69
Total effect	0.760	0.591	0.969	.029	1.292	1.033	1.619	.026	1.405	1.120	1.770	.004

PNDs, postnatal days; CI, confidence interval; PGF, postnatal growth failure.

^a^It is assumed that gestational age, Apgar score at five min, small for gestational age, antenatal steroid use, and presence of pregnancy-induced hypertension, intraventricular hemorrhage (≥ grade 3), total sepsis, periventricular leukomalacia, bronchopulmonary dysplasia (≥ moderate), necrotizing enterocolitis (≥ 2b), and retinopathy of prematurity requiring laser therapy are sufficient to control for confounding.

^b^95% confidence intervals are constructed using the bootstrapping method based on 1000 resamples with replacement.

^c^Mediation analysis was performed using the imputation-based natural effect models of the medflex package with P2 as reference.

## Discussion

In this study, we demonstrated that the influence of TFF on VLBW infants during NICU stay persisted after controlling for all variables known at birth or identified during NICU stay and was significantly associated with their survival without NDD and long-term PGF at 18–24 months’ CA. Specifically, TFF ≤ 15 PNDs in VLBW infants was associated with a lower risk of motor domain NDD and weight and height PGF than was TFF > 15 PNDs. In contrast, delayed TFF (> 45 PNDs) was significantly associated with a higher NDD risk for motor, mental, and social domains and height and head-circumference PGF than was earlier TFF < 31 PNDs. Furthermore, we revealed that long-term PGF did not mediate the association between delayed TFF and increased NDD risks for VLBW infants at 18–24 months’ CA. All these suggest that TFF is independently associated with long-term neurodevelopment without mediation by long-term postnatal growth in VLBW infants.

Apart from the suggested benefits of early enteral nutrition on modulation of the gut microbiota, metabolites, and immune function^43,44^, it was expected that better postnatal growth would be strongly associated with better neurodevelopment in cases of early TFF in VLBW infants. Furthermore, delayed TFF is associated with an increased extra-uterine growth retardation incidence at discharge^4,5,45^ and increased risk of poor neurodevelopment^12^. However, data regarding this association is conflicting. Furthermore, extra-uterine growth restriction categorization at an approximate term was not associated with abnormal neurodevelopment^16^. In a previous study, an earlier enteral feeding strategy to get an earlier TFF was not associated with long-term growth improvement at 2 years in extremely-low-birth-weight infants^11^. A recent prospective study observed no difference in 2-year neurodevelopment between VLBW infants achieving early TFF and those in the slower enteral feeding advancement group with slightly delayed TFF^13^. However, in both studies^11,45^, TFF of rapid and slow feeding groups fell into P1 group of our study. Nonetheless, it might be too early to demonstrate the effect of early enteral transition on long-term growth or neurodevelopmental outcomes. Many observational studies have revealed a positive correlation between postnatal growth and neurodevelopmental outcomes^46–48^. However, interventional studies that aimed to promote postnatal growth in preterm infants did not reveal consistent beneficial effects of faster postnatal growth on subsequent neurodevelopment^15,49^. This discrepancy between observational and interventional studies might be derived from the confounding effects of various neonatal morbidities of infants, affecting both long-term growth and neurodevelopment, which should be considered more in observational studies.

In this study, we revealed a firm association between TFF and long-term neurodevelopment and between TFF and long-term growth after adjusting for confounding factors, such as significant neonatal morbidities. We also demonstrated, for the first time to our knowledge, that long-term PGF did not mediate this association between TFF and long-term neurodevelopment.

TFF is inextricably related to immaturity and related illnesses, including short-term morbidities, and is thus regarded as an index of the clinical course for VLBW infants during NICU stay. Nonetheless, there are significant variations in clinical practice^50^ concerning determination of TFF, such as when the enteral feeds were initiated^51,52^, how fast they advanced^9,44,45,53,54^ types of milk that were provided^55,56^, and how feeding intolerance was managed by the medical staff^57^ in each unit. Therefore, compelling evidence on the positive impact of early TFF during NICU stay on long-term neurodevelopmental outcomes, which is independent of and not mediated by long-term growth, would provide an important rationale for quality improvement strategies targeting early TFF in VLBW infants during neonatal intensive care.

In this study, the median TFF in all VLBW infants was far more delayed than those reported in Europe, Oceania, and North America (18 PNDs *versus* 8–15 PNDs)^2^. This study comprised a multicenter cohort that covers over 80% of VLBW infants in Korea^17^, including individual centers that may have different feeding protocols and different qualities of care. Therefore, systemic quality improvement initiatives, such as encouraging enteral feeding with breast milk rather than formula in NICU^58^ and implementing targeted feeding protocols for individuals in NICU^4^ are necessary to reduce TFF in VLBW infants.

This study has several limitations. First, we could not identify individual NICU practices that affected TFF, including types of milk provided, feeding strategy, and information regarding parenteral nutrition, which might affect important nutritional status. Second, the lack of information regarding feeding strategies of individual institutes and inability to exclude unknown factors affecting growth and neurodevelopment should be considered. These might suggest that residual confounding is more likely than a true association, implying that smaller and sicker infants have delayed feeding and worse outcomes. Third, as 61% of the enrolled infants were followed up at 18–24 months’ CA, there was a possible bias due to loss-to-follow-up. Among the infants followed up, 73.3% were evaluated for neurodevelopmental outcomes, with 42.4% undergoing Bayley tests, which might decrease the strength of results. Finally, as K-DST and K-ASQ are parent-completed questionnaires, they had recall bias limitations, although these developmental screening tools were valid and correlated with BSID-II^33,34^.

However, the strength of this study is that, to our knowledge, it is the first to systemically evaluate an independent association between enteral feeding completion, indicated as TFF, and long-term neurodevelopment with mediation analysis including long-term growth in a large prospective cohort using data collected with uniform criteria and of well-supervised quality^17^.

## Conclusion

Our study showed a strong association between TFF and long-term neurodevelopment in VLBW infants. Long-term PGF did not mediate the correlation between TFF and long-term neurodevelopment in VLBW infants. Therefore, our results suggest that TFF directly impacts survival without NDD. Consequently, these findings provide a rationale for translating the achievement of early TFF into quality improvement management of VLBW infants.

## Supplementary Information

Supplementary Information.

## Data Availability

The data that support the findings of this study are available from the corresponding author (yunsil.chang@gmail.com) upon reasonable request.
