# Supplementary material for: Impact of time to full enteral feeding on long-term neurodevelopment without mediating by postnatal growth failure in very-low-birth-weight-infants
Source: Sci Rep. 2023 Feb 20;13:2990. doi: 10.1038/s41598-023-29646-1 (PMC9941577; doi:10.1038/s41598-023-29646-1)
Supplement: Supplementary file 1 — Supplementary Information. [file 41598_2023_29646_MOESM1_ESM.pdf]

Impact of time to full enteral feeding on long-term neurodevelopment without mediating postnatal growth failure in very-low-birth-weight-infants

Shin Ae Yoon[1¶], Myung Hee Lee[2¶], Yun Sil Chang[3,4,5*]

[1]Department of Pediatrics, Chungbuk National University Hospital, Chungbuk National University School of Medicine, Cheongju, Korea

[2]Research & Statistical Center, Social Information Research Institute, Seoul, Korea

[3]Department of Pediatrics, Samsung Medical Center, Sungkyunkwan University School of Medicine, Seoul, Korea

[4]Department of Health Sciences and Technology, SAIHST, Sungkyunkwan University, Seoul, Korea

[5]Samsung Medical Center, Cell and Gene Therapy Institute, Seoul, Korea

[¶] These authors contributed equally to this work.

Supplementary data

Supplementary Table S1. Details of the Results of Neurodevelopmental Tests in This Cohort

		BSID-II or III score		
		≥ 70 (n = 884)	< 70 (n = 270)	Missing (n = 1568)
K-ASQ or K-DST score	≥ -2SD (n = 1180)	Normal (n = 462)	Abnormal (n = 65)	Normal (n = 653)
K-ASQ or K-DST score	< -2SD (n = 344)	Normal (n = 81)	Abnormal (n = 101)	Abnormal (n = 162)
K-ASQ or K-DST score	Missing (n = 1198)	Normal (n = 341)	Abnormal (n = 104)	Missing (n = 753)

Abbreviations: BSID, Bayley Scales of Infant and Toddler Development; K-ASQ, Korean version of the Ages and Stages Questionnaire; K-DST, Korean-Developmental Screening Test for infants and children

Supplementary Table S2. Demographic Characteristics and Short-term Outcomes

**Variables**	**P1: <16**[a] **(n = 2003)**	**P2: 16-30**[b] **(n = 1301)**	**P3: 31-45**[c] **(n = 599)**	**P4: >46**[d] **(n = 554)**	**Total (n = 4457)**	***P*-value**
Gestational age, mean (SD), weeks	29.6 (1.8)	28.2 (2.1)	27.4 (2.1)	26.6 (2.0)	28.5 (2.2)	<.001[d<c<b<a]
Birth weight, mean (SD), g	1218.2 (210.0)	1049.7 (248.7)	977.9 (242.7)	865.5 (232.3)	1093.1 (261.4)	<.001[d<c<b<a]
Male sex, No. (%)	973 (48.6)	671 (51.6)	323 (53.9)	281 (50.7)	2248 (50.4)	.10
One-minute Apgar score, mean (SD)	5.2 (1.8)	4.5 (1.9)	4.0 (1.)8	3.6 (1.8)	4.7 (1.9)	<.001[d<c<b<a]
Five-minute Apgar score, mean (SD)[e]	7.3 (1.4)	6.7 (1.7)	6.3 (1.7)	6.0 (1.9)	6.9 (1.7)	.001[d<c<b<a]
Cesarean section, No. (%)	1,503 (75.0)	990 (76.1)	457 (76.3)	419 (75.6)	3369 (75.6)	.88
Small for gestational age, No. (%)	320 (16.0)	243 (18.7)	99 (16.5)	106 (19.1)	768 (17.2)	.13
Antenatal steroids, No. (%)	1,581 (79.7)	1,025 (79.8)	471 (80.2)	458 (85.3)	3535 (80.5)	.03
PROM ≥ 24 hours, No. (%)	545 (27.4)	340 (26.2)	153 (25.8)	157 (28.5)	1195 (27.0)	.67
Histological chorioamnionitis, No. (%)	545 (32.4)	382 (34.9)	189 (36.7)	200 (43.1)	1316 (35.0)	<.001
PIH, No. (%)	387 (19.3)	226 (17.4)	87 (14.5)	78 (14.1)	778 (17.5)	.005
Maternal diabetes mellitus, No. (%)	22 (1.1)	10 (0.8)	6 (1.0)	5 (0.9)	43 (1.0)	.82
Maternal age, mean (SD), years	32.6 ± 4.2	32.9 ± 4.1	32.9 ± 4.3	33.0 ± 4.0	32.8 ± 4.2	0.18

Parity (≥1), No. (%)	770/2003 (38.4)	542/1301 (41.6)	253/599 (42.2)	213/554 (38.4)	1778/4457 (39.8)	0.15
Maternal high education, No. (%)	1197/1633 (73.3)	756/1047 (72.2)	354/483 (73.3)	310/415 (74.7)	2617/3578 (73.1)	0.80
Paternal high education, No. (%)	926/1193 (77.6)	603/790 (76.3)	286/371 (77.1)	224/310 (72.3)	2039/2664 (76.5)	0.31
Maternal Koreans, No. (%)	1936 (96.6)	1255 (96.4)	579 (96.6)	530 (95.6)	4300 (96.5)	0.73
Paternal Koreans, No. (%)	1968 (98.2)	1279 (98.3)	586 (97.8)	538 (97.1)	4371 (98.1)	0.31
**Short-term outcomes**						
Mortality, No. (%)	19 (0.9)	11 (0.8)	13 (2.2)	20 (3.6)	63 (1.4)	<.001
Cause of death, No./total No. (%)						
Cardiorespiratory[e]	6/19 (32)	4/11 (36)	4/13 (31)	8/20 (40)	22/63 (35)	.95
Gastrointestinal	8/19 (42)	5/11 (46)	4/13 (31)	6/20 (30)	23/63 (37)	
Infection	2/19 (11)	1/11 (9)	4/13 (31)	3/20 (15)	10/63 (16)	
Others	3/19 (16)	1/11 (9)	1/13 (8)	3/20 (15)	8/63 (13)	
Postnatal days at death, median (IQR)[f]	35 (21-45)	38 (34–143)	63 (38–228)	121.5 (93–194.5)	64 (35–164)	<.001 [a<b<c<d]
IVH (≥ grade 3), No. (%)	31 (1.5)	99 (7.6)	83 (13.9)	100 (18.0)	313 (7.0)	<.001
Sepsis, total, No. (%)	167 (8.3)	273 (21.0)	209 (34.9)	293 (52.9)	942 (21.1)	<.001
NEC (≥ stage 2), No. (%)	56 (2.8)	53 (4.1)	53 (8.9)	73 (13.2)	235 (5.3)	<.001

PVL, No. (%)	105 (5.2)	101 (7.8)	79 (13.2)	91 (16.5)	376 (8.4)	<.001
BPD (≥ moderate), No. (%)	282 (14.2)	423 (32.7)	272 (46.1)	410 (74.3)	1387 (31.4)	<.001
ROP requiring laser treatment, No. (%)	56 (2.8)	116 (9.0)	105 (17.7)	152 (27.4)	429 (9.7)	<.001
Duration of PN, mean (SD), day[f]	12.0 (11.4)	27.1 (14.6)	42.0 (16.1)	69.7 (33.0)	27.6 (25.5)	<.001 [a<b<c<d]
TFF, mean (SD), day	9.3 (3.6)	22.1 (4.2)	37.2 (4.3)	65.1 (21.7)	23.7 (20.0)	<.001 [a<b<c<d]
TFF, median (IQR), day	9 (6-12)	22 (18–25)	37 (33–41)	59 (52–72)	18 (10–31)	
Hospital stays, mean (SD), day[f]	57.9 (25.4)	77.8 (32.8)	90.5 (42.2)	113.2 (46.0)	75.0 (38.1)	<.001 [a<b<c<d]

Abbreviations: PROM, premature rupture of membrane; PIH, pregnancy-induced hypertension; IQR, interquartile range; PDA, patent ductus arteriosus; IVH, intraventricular hemorrhage; NEC, necrotizing enterocolitis; BPD, bronchopulmonary dysplasia; PVL, periventricular leukomalacia; ROP, retinopathy of prematurity; PN, parenteral nutrition; TFF, time to achieve full enteral feeding; IQR, interquartile range; SD, standard deviation

[e] A Fisher exact test is performed instead of the Pearson's chi-squared test because the expected frequency in the contingency table is less than 5.

[f] Because the assumptions of one-way ANOVA are not met, the Kruskal–Wallis test and post-hoc test (Dunn test) are conducted.

Supplementary Table S3. Primary and Secondary Outcomes at 18–24 Months′ Corrected Age

Variables	P1: ≤15[a] (n = 1145)	P2: 16–30[b] (n = 816)	P3: 31–45[c] (n = 399)	P4: ≥46[d] (n = 362)	*P*-value
**Primary outcomes**					
Survival, N (%)	1141 (99.6)	815 (99.8)	399 (100.0)	361 (99.7)	.54
Survival without neurodevelopmental delay	1041/1141(91.2)	684/815 (83.9)	303/399 (75.9)	248/361 (68.7)	<.001
**Secondary outcomes**					
Cerebral palsy, No./total No. (%)	40/1124 (3.6)	59/793 (7.4)	48/387 (12.4)	43/344 (12.5)	<.001
Visual impairment	112/1089 (10.3)	124/769 (16.1)	84/373 (22.5)	99/342 (28.9)	<.001
Deafness, No./total No. (%)	14/973 (1.4)	17/675 (2.5)	13/324 (4.0)	11/297 (3.7)	.02
Neurodevelopmental delay in					
Total domain, No./total No. (%)	100/769 (13.0)	131/613 (21.4)	96/299 (32.1)	113/288 (39.2)	<.001
Motor domain, No./total No. (%)	57/769 (7.4)	90/613 (14.7)	78/299 (26.1)	88/288 (30.6)	<.001
Mental domain, No./total No. (%)	71/769 (9.2)	85/613 (13.9)	66/299 (22.1)	79/288 (27.4)	<.001
Social domain, No./total No. (%)	18/601 (3.0)	24/469 (5.1)	31/231 (13.4)	37/223 (16.6)	<.001
**Others (Abnormal neurodevelopmental outcomes)**					
Bayley II test (n=713)					
MDI, mean (SD)	88.5±19.6	84.7±21.9	84.1±21.4	76.8±21.3	<.001[d<a, b]
MDI <70, No./total No. (%)	42/266 (15.8)	55/227 (24.2)	29/115 (25.2)	38/105 (36.2)	<.001

PDI, mean (SD)	88.9±19.2	84.3±20.6	81.8±20.9	78.1±18.8	<.001[c,d<a]
PDI <70, No./total No. (%)	37/266 (13.9)	49/227 (21.6)	34/115 (29.6)	32/105 (30.5)	<.001
Bayley III test (n = 441)					
Cognitive, mean (SD)	101.1±14.0	96.9±15.2	92.0±16.4	87.6±16.8	<.001[c,d<a ,b]
Cognitive <70, No./total No. (%)	4/180 (2.2)	6/133 (4.5)	5/56 (8.9)	14/72 (19.4)	<.001
Language, mean (SD)	97.1±15.1	94.7±15.7	89.9±15.5	85.5±15.5	<.001[c,d<a, b]
Language <70, No./total No. (%)	7/180 (3.9)	3/133 (2.3)	5/56 (8.9)	10/72 (13.9)	.004
Motor, mean (SD)	99.7±13.6	94.6±16.1	91.5±18.8	82.7±19.7	<.001[d<c<a, b]
Motor <70, No./total No. (%)	3/180 (1.7)	12/133 (9.0)	7/56 (12.5)	20/72 (27.8)	<.001
K-ASQ (n = 363), No./total No. (%)					
Communication < -2 SD	13/164 (7.9)	12/98 (12.2)	11/50 (22.0)	12/51 (23.5)	.007
Gross motor < -2 SD	6/164 (3.7)	19/98 (19.4)	10/50 (20.0)	20/51 (39.2)	<.001
Fine motor < -2 SD	6/164 (3.7)	7/98 (7.1)	4/50 (8.0)	9/51 (17.7)	.01
Problem-solving < -2 SD	8/164 (4.9)	9/98 (9.2)	10/50 (20.0)	9/51 (17.7)	.003
Personal/social < -2 SD	7/164 (4.3)	11/98 (11.2)	9/50 (18.0)	11/51 (21.6)	.001
K-DST (n = 1276), No./total No. (%)					
Gross motor < -2 SD	30/469 (6.4)	53/411 (12.9)	51/209 (24.4)	48/187 (25.7)	<.001
Fine motor < -2 SD	12/469 (2.6)	20/411(4.9)	29/209 (13.9)	35/187 (18.7)	<.001

Cognition < -2 SD	17/469 (3.6)	20/411 (4.9)	27/209 (12.9)	29/187 (15.5)	<.001
Language < -2 SD	26/469 (5.5)	26/411 (6.3)	26/209 (12.4)	23/187 (12.3))	.001
Sociality < -2 SD	11/469 (2.3)	14/411 (3.4)	23/209 (11.0)	27/187 (14.4)	<.001
Self-help < -2 SD	13/466 (2.8)	23/410 (5.6)	28/209 (13.4)	32/185 (17.3)	<.001

Abbreviations: MDI, mental developmental index; PDI, psychomotor developmental index; K-ASQ, Korean version of the Ages and Stages Questionnaire; K-DST, Korean-Developmental Screening Test for infants and children; SD, standard deviation;

[e] A Fisher′s exact test is performed instead of the Pearson's chi-squared test because the expected frequency in the contingency table is less than 5.

Supplementary Table S4. Change in Growth Outcomes at Birth, Discharge, and 18–24 Months′ Corrected Age

	**P1: ≤ 15[a]** (n = 1145)	**P2: 16-30[b]** (n = 816)	**P3: 31-45[c]** (n = 399)	**P4: 46 ≤[d]** (n = 362)	***P*-value[e]**
**Weight Z-Score**					
At birth	-0.27±0.83	-0.18±0.92	-0.06±0.95	-0.17±0.99	$<.001^{a<b=d<c}$
At discharge	-1.32±0.98	-1.59±1.08	-1.75±1.18	-2.28±1.26	$<.001^{d<c<b<a}$
During 18–24 months	-0.19±1.12	-0.40±1.22	-0.46±1.32	-0.88±1.28	$<.001^{d<c=b<a}$
Difference between birth and discharge	-1.05±0.76	-1.41±0.90	-1.71±1.03	-2.14±1.09	$<.001^{d<c<b<a}$
Difference between discharge and the CA of 18–24 months	1.12±1.21	1.21±1.25	1.36±1.33	1.49±1.37	$<.001^{a=b<c=d}$
Postnatal growth failure at discharge[f]	416/1990 (20.9)	411/1276 (32.2)	205/572 (35.8)	268/498 (53.8)	$<.001$
Postnatal growth failure at a CA of 18–24 months[f]	64/1145 (5.6)	84/815 (10.3)	52/398 (13.1)	61/361(16.9)	$<.001$
**Height Z-Score**					
At birth	-0.21±1.07	-0.19±1.14	-0.11±1.18	-0.26±1.26	.08
At discharge	-1.54±1.24	-1.95±1.34	-2.20±1.59	-2.79±1.63	$0.001^{d<c<b<a}$
During 18–24 months	-0.13±1.23	-0.33±1.32	-0.42±1.59	-0.81±1.51	$0.001^{d<c=b<a}$
Difference between birth and Discharge	-1.32±1.13	-1.76±1.19	-2.07±1.40	-2.48±1.38	$0.001^{d<c<b<a}$
Difference of discharge and the CA of 18–24 months	1.41±1.40	1.71±1.51	1.88±2.01	2.01±1.72	$0.001^{a<b=c<d}$

Postnatal growth failure at discharge[f]	559/1788 (31.3)	521/1145 (45.5)	271/508 (53.3)	284/422 (67.3)	0.001
Postnatal growth failure at a CA of 18-24 months[f]	59/1029 (5.7)	79/745 (10.6)	39/362 (10.8)	63/341 (18.5)	0.001
**Head Circumference Z-Score**					
At birth	-0.08±1.08	-0.11±1.17	-0.08±1.23	-0.25±1.12	.028 [d<c=b=a]
At discharge	-0.93±1.08	-1.24±1.10	-1.50±1.24	-2.03±1.30	0.001[d<c<b<a]
During 18–24 months	-0.11±1.36	-0.27±1.46	-0.58±1.79	-1.08±1.76	0.001[d<c<b=a,c=b]
Difference between birth and discharge	-0.83±1.19	-1.11±1.24	-1.41±1.43	-1.76±1.27	0.001[d<c<b<a]
Difference between discharge and the CA of 18-24months	0.82±1.36	0.97±1.49	0.85±1.72	0.98±1.51	.25
Postnatal growth failure at discharge[f]	225/1866 (12.1)	264/1202 (21.9)	151/530 (28.5)	225/443 (50.8)	0.001
Postnatal growth failure at a CA of 18-24 months[f]	87/854 (10.1)	74/636 (11.6)	63/320 (19.6)	81/289 (28.0)	0.001

Abbreviations: CA, corrected age

[e] According to the assumption of normality, a one-way ANOVA or Kruskal-Wallis test is conducted.

[f] Postnatal growth failure is defined as a Z-score <–2.

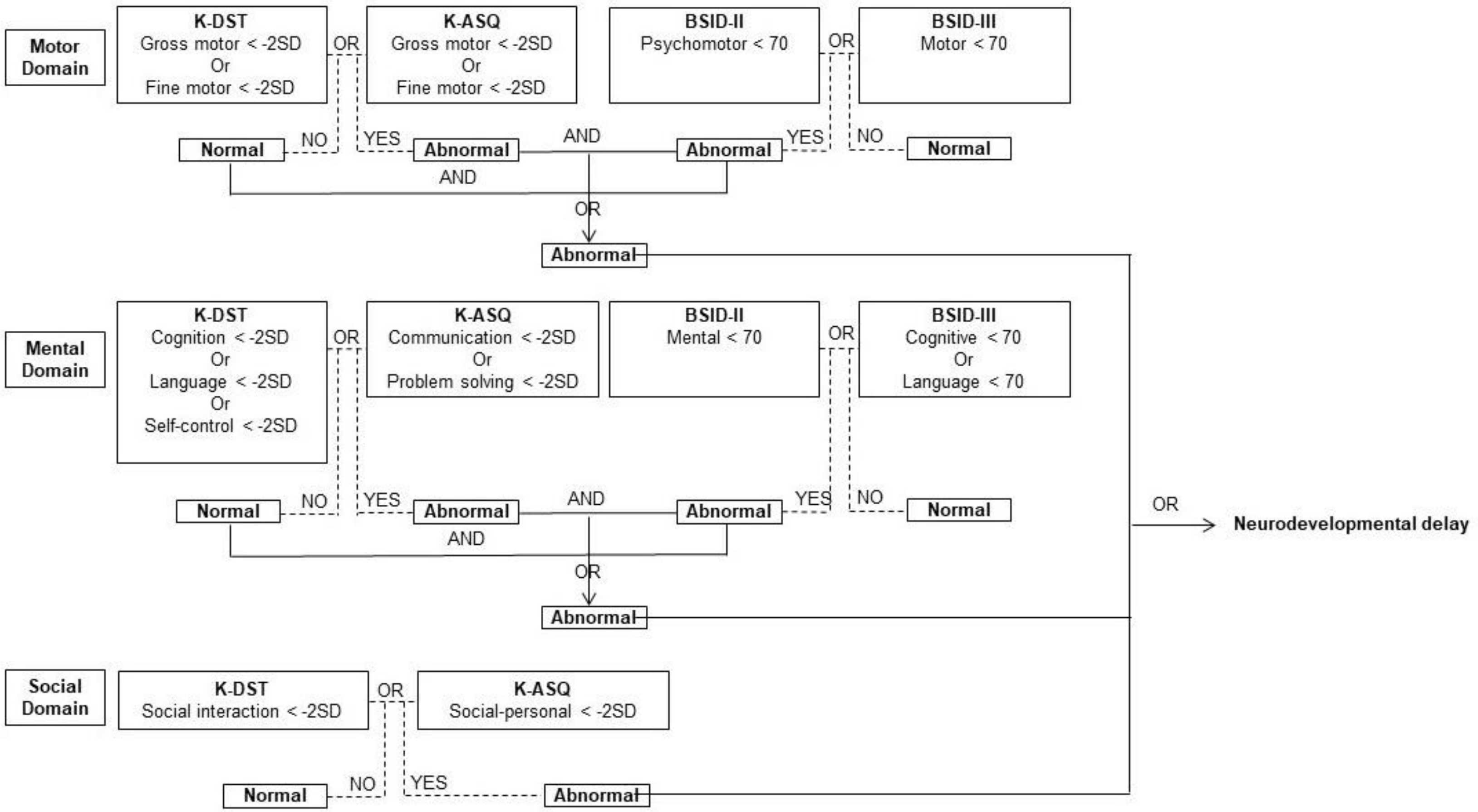

Supplementary Figure S1. Neurodevelopmental delay domain framework at 18–24 months' corrected age

Abbreviations: K-DST, Korean Developmental Screening Test for infants and children; K-ASQ, Korean version of the Ages and Stages Questionnaire; BSID, Bayley Scales of Infant and Toddler Development; SD, standard deviation
